# Optimization of Supercritical Fluid CO_2_ Extraction from Yellow Horn Seed and Its Anti-Fatigue and Antioxidant Activity

**DOI:** 10.3390/molecules28124853

**Published:** 2023-06-19

**Authors:** Siyan Lyu, Haoran Wang, Tingjun Ma

**Affiliations:** College of Food Science and Engineering, Beijing University of Agriculture, Beijing 102206, China; lsyan9885@163.com (S.L.); wanghaoran010@126.com (H.W.)

**Keywords:** yellow horn oil 1, supercritical fluid carbon dioxide extraction 2, anti-fatigue 3, antioxidant

## Abstract

A supercritical fluid carbon dioxide (SF-CO_2_) extraction method was used to obtain the optimum process for extracting yellow horn seed oil. The anti-fatigue and antioxidant properties of the extracted oil were investigated through animal experiments. The optimum process conditions for SF-CO_2_ extraction of the yellow horn oil were 40 MPa, 50 °C and 120 min, with an extraction yield of 31.61%. The high-dose group of yellow horn oil could significantly increase the weight-bearing swimming time, the hepatic glycogen (HG) content and decrease the lactic acid (LA) content and blood urea nitrogen (BUN) content (*p* < 0.05) in mice. Moreover, it improved the antioxidant ability by reducing the malondialdehyde (MDA) content (*p* < 0.01) and raising the glutathione reductase (GR) content and superoxide dismutase (SOD) content (*p* < 0.05) in mice. Yellow horn oil has the effects of being an anti-fatigue and antioxidant substance, which provides a basis for its further utilization and development.

## 1. Introduction

Yellow horn (*Xanthoceras sorbifolia*), a woody deciduous shrub or small tree in the Sapindaceae family, is an important bioenergy feedstock in China as an alternative to petroleum energy [1]. It is native to China and has now also been introduced worldwide for the development of biomass energy sources. Yellow horn mainly grows in the north of China, such as Inner Mongolia Autonomous Region, Shanxi and Gansu Province, and it is also cultivated in Ningxia, Shandong, Hebei and Liaoning Province [2]. It is resistant to cold, drought and barren conditions [3]. Yellow horn seed contains 55–70% oil [4], which is a good edible semi-dry oil with yellow color, an aromatic and tasty smell, low acidity, a high iodine value suitable for long-term storage and is highly transparent, so it enjoys a high reputation in the international market. Yellow horn oil consists of 93% unsaturated fatty acids, including about 40% linoleic acid, 30% oleic acid and 3% nervonic acid [5,6]. Unsaturated fatty acids could regulate blood lipids, lower blood pressure, prevent atherosclerosis, improve lung function, treat respiratory diseases, modulate the immune system, improve arthritis symptoms and reduce pain [7]. Nervonic acid can repair damaged brain nerves, thus preventing brain nerve aging and keeping them active [8]. It also contains a variety of active substances such as flavonoids and polyphenols [9]. Therefore, yellow horn oil plays a role in preventing disease.

Currently, methods for extracting oil from plant seeds include solvent extraction [10], aqueous enzymatic extraction [11] and mechanical pressing. However, from the perspectives of extraction efficiency, product purity and solvent residue problems, SF-CO_2_ extraction technology has the advantages of easy operation, low energy consumption, high extraction efficiency and high product purity [12,13], and has been applied in the fields of biopharmaceuticals and food, especially for oil extraction. Although a study on yellow horn oil by SF-CO_2_ extraction has been reported [4], the optimization of SF-CO_2_ extraction conditions is limited.

Overexertion is a state of sub-health due to excessive working hours, labor intensity and psychological stress leading to exhaustion [14]. Its greatest risk is to cause rapid deterioration of latent diseases [15]. For example, it leads to acute circulatory organ disorders such as cerebrovascular disease or cardiovascular disease, and even causes fatal symptoms. Sudden death is also known as “death from overwork”. So, it is very beneficial to improve the situation of an overworked body through our daily diet. The current research focuses on the antioxidant properties of yellow horn oil. A similar study in the literature has examined the antioxidant activity of yellow horn seed oil, which was assessed by a 2,2-diphenyl-1-trinitrophenylhydrazine (DPPH) radical scavenging assay and a β-carotene bleaching assay [3]. Moreover, Li et al. [16] found triterpene saponins extracted from the residue of yellow horn oil extraction which had significant free radical scavenging activity. However, there have been no reported studies on the anti-fatigue effect of yellow horn oil.

In this experiment, yellow horn oil was obtained by SF-CO_2_ extraction, and the optimized process conditions from the aspects of extraction time, extraction temperature and extraction pressure were analyzed. We determined the fatty acids of the yellow horn oil extracted under the optimal conditions. Then, the mice were fed the oil under optimal conditions, and its anti-fatigue and antioxidant activities were investigated by a weight-bearing swimming test and a physiochemical analysis of the mice, respectively. Through the research, it is hoped that yellow horn oil can be used in pharmaceutical, cosmetic and functional food applications to increase its value.

## 2. Results

### 2.1. Influence of SF-CO_2_ Extraction Conditions on the Yield of Yellow Horn Oil

**Figure 1:**
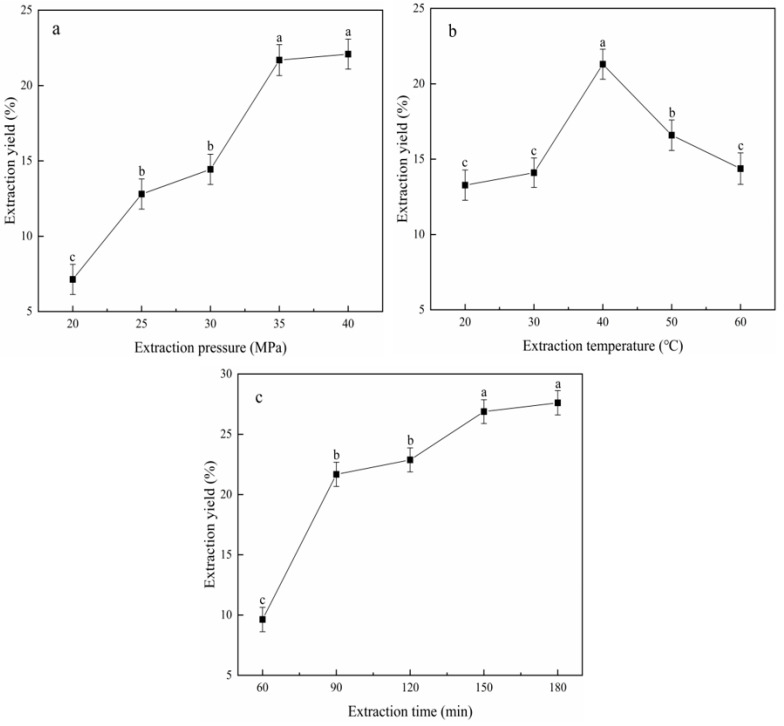
Effect of extraction pressure (**a**), extraction temperature (**b**) and extraction time (**c**) on extraction yield. Note: different lowercase letters in the same column indicate significant differences (*p* < 0.05).

The extraction pressure was set at 20, 25, 30, 35 and 40 MPa, the extraction time was 90 min and the extraction temperature was 40 °C for the test, and the results are shown in Figure 1a. The extraction temperature was set to 20, 30, 40, 50 and 60 °C, the extraction pressure was chosen to be 35 MPa and the extraction time was 90 min for the test, and the results are shown in Figure 1b. The extraction time was set to 60, 90, 120, 150 and 180 min, the extraction temperature was 40 °C and the extraction pressure was 35 MPa for the test, and the results are shown in Figure 1c.

As can be seen from Figure 1a, the extraction yield tended to increase when the extraction pressure was between 20 and 35 MPa. When the pressure exceeded 35 MPa, the extraction yield changed slowly. This is consistent with the findings of Jokić’s study [17]. Therefore, considering the cost and other factors, the most suitable extraction pressure was chosen as 35 MPa, and the extraction pressure of 30 MPa was selected as the upper level and 40 MPa as the lower level for the orthogonal design test.

From Figure 1b, it can be seen that the extraction yield gradually increased from 20 to 40 °C until it reached a peak of 21.31% at 40 °C and gradually decreased above 40 °C. This trend is similar to the results in the literature [18]. Therefore, 40 °C was selected as the most suitable extraction temperature, and the upper level of 30 °C and the lower level of 50 °C were selected as the extraction temperatures for the orthogonal design test.

From Figure 1c, it can be seen that the extraction yield increased during the extraction time of 60–150 min. In the interval of 150–180 min, the extraction yield of the yellow horn oil gradually increased slowly and tended to level off. It is in agreement with the work of Liu et al. [19]. Considering the energy consumption and other factors, 150 min was chosen as the optimum extraction time, and 120 min was chosen as the upper level and 180 min as the lower level for the orthogonal factorial design test.

### 2.2. Optimization of Conditions for SF-CO_2_ Extraction of Yellow Horn Oil

According to the extreme difference (*R*) in Table 1a, it can be seen that *A* > *C* > *D* > *B,* indicating extraction pressure > extraction time > extraction temperature. From the visual analysis, it can be seen that the best process condition was *A*_3_*B*_3_*C*_2_, representing a pressure of 40 MPa, temperature of 50 °C and time of 120 min, with an extraction yield of 31.61%. From the results of the ANOVA (Table 1b), it can be seen that *A* (extraction pressure) and *C* (extraction time) had a significant effect on the experiment, and *B* (extraction temperature) was not significant and had the least relative effect among the factors examined. A similar conclusion was obtained in SF-CO_2_ extraction on hemp cake [20]. The fatty acid composition and content of yellow horn oil were determined under optimal extraction conditions. As seen in Table 2, yellow horn oil contained mainly oleic and linoleic acids with 33.40% and 44.81%, respectively. In addition, it also contained 4.06% of nervonic acid, which was consistent with the study by Huang [9] et al. This indicated that the SF-CO_2_ extraction did not change the fatty acid composition of the yellow horn oil.

### 2.3. Anti-Fatigue Test with Extracted Yellow Horn Oil

**Figure 2:**
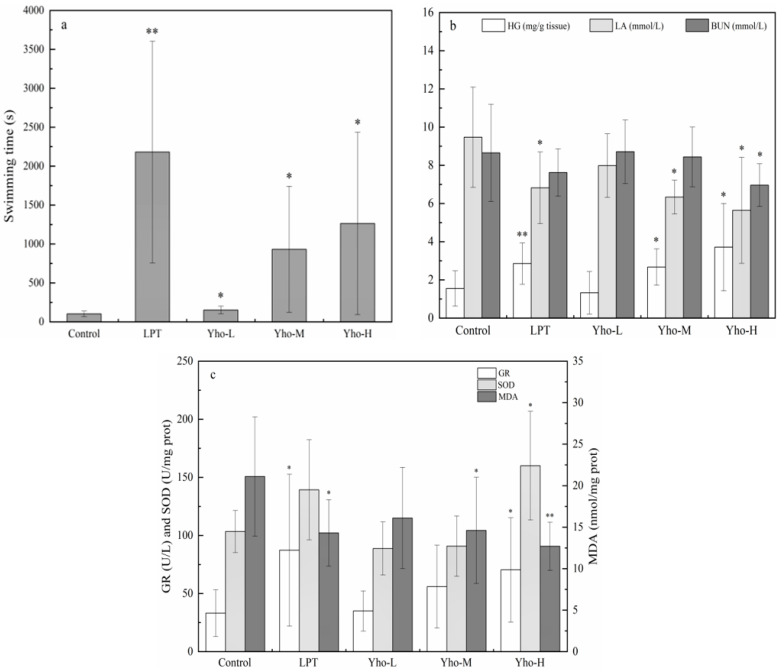
Effect of yellow horn oil on weight-bearing swimming time in mice (**a**). Effect of yellow horn oil on the content of HG (hepatic glycogen), LA (lactic acid) and BUN (blood urea nitrogen) in liver tissue of mice (**b**). Effect of yellow horn oil on serum GR (glutathione reductase) levels and SOD (superoxide dismutase), MDA (malondialdehyde) activities in mice (**c**). Note: Compared with the control group, * *p* < 0.05, ** *p* < 0.01. Blank control group (Control), lentinan polysaccharide tablet (LPT), yellow horn oil low-dose group (Yho-L), yellow horn oil medium-dose group (Yho-M), yellow horn oil high-dose group (Yho-H).

The endurance of the mice, meaning the ability to resist fatigue, could be measured by the weight-bearing swimming time of the mice. As can be seen from Figure 2a, Yho-L, Yho-M and Yho-H all significantly prolonged the weight-bearing swimming time in mice compared to the blank control group (*p* < 0.05). The positive control group was the most effective in prolonging the weight-bearing swimming time in mice (*p* < 0.01). Although the yellow horn oil was not as good as the LPT in improving fatigue tolerance in mice, it showed a significant effect on prolonging the weight-bearing swimming time.

As shown in Figure 2b, Yho-M and Yho-H significantly increased the content of HG in the liver tissue of mice compared to the blank control (*p* < 0.05). LPT increased the HG content more significantly (*p* < 0.01); LPT, Yho-M and Yho-H all significantly (*p* < 0.05) reduced serum LA levels in mice. LPT, Yho-L, Yho-M and Yho-H all reduced the serum BUN content in mice to different degrees, with Yho-H significantly reducing the BUN content (*p* < 0.05), and the effect was better than LPT.

### 2.4. Antioxidant Activity Test with Extracted Yellow Horn Oil

It can be seen from Figure 2c that LPT, Yho-L, Yho-M and Yho-H all increased the GR content in mice serum compared with the blank control group, in which LPT and Yho-H could significantly increase the GR content (*p* < 0.05). LPT and Yho-H both increased SOD activity in liver tissues of mice, and the high dose of the yellow horn oil significantly increased SOD activity (*p* < 0.05). LPT, Yho-L, Yho-M and Yho-H all reduced MDA activity in liver tissues of mice, with LPT and Yho-M significantly reducing MDA activity (*p* < 0.05) and Yho-H highly significantly reducing MDA activity (*p* < 0.01). Yho-H reduced MDA activity more significantly (*p* < 0.01), and it is worth noting that Yho-H increased SOD activity and reduced MDA activity better than LPT.

## 3. Discussion

The extraction yield of yellow horn oil increased with the growth of the extraction pressure. However, when it exceeded a certain range, the extraction yield increased slowly until it did not change. The reason was that when the pressure increased within a certain range, the density of CO_2_ also increased, resulting in an increase in the solubility of the yellow horn oil in the CO_2_ fluid. However, when the pressure was higher than a certain range, the CO_2_ was severely compressed, which in turn strengthened the rejection between the oil and the CO_2_ fluid [21]. So, the yield rose slowly. As the extraction temperature rose, the extraction yield of yellow horn oil tended to increase. When a certain threshold was exceeded, the extraction yield fell. This was due to the fact that when the temperature gradually increased, it would intensify the intermolecular movement, which increased the mass transfer rate and promoted the diffusion of the yellow horn oil in the CO_2_ fluid. However, when the temperature kept increasing, the density of CO_2_ reduced and the solubility of the yellow horn oil decreased, thus reducing the oil yield [22]. In addition, at higher extraction temperatures, the contact of CO_2_ with the seeds was further reduced with the decrease in CO_2_ density [23]. With the increase in the extraction time, the extraction yield of yellow horn oil increased. However, when the CO_2_ was in full contact with the oil, the extraction yield could not increase more. This was because at the beginning of the experiment, the extraction yields increased with time as the oil was in contact with the CO_2_ fluid. When the solvent and solute reached equilibrium, the extraction yields leveled off. After the optimization of the orthogonal test, the extraction yield was 31.61% under the extraction conditions of 40 MPa, 50 °C and 120 min. The extraction rate of yellow horn oil in this study was lower than that of Gu et al. [4]. This may be due to the fact that yellow horn is produced from different regions resulting in different oil contents, thus also indicating that the variety of raw material is a very important factor. It has been shown in the literature that the final yield of yellow horn seed oil was 48.6% using a microwave-assisted method with water extraction under acidic conditions. Although the yield was high, the acidic condition was regulated by HCl, so the safety of yellow horn oil could not be guaranteed. Moreover, the SF-CO_2_ method is a green and high-performance extraction technology that is environmentally sustainable [24]. Yellow horn oil was obtained under optimal extraction conditions and its fatty acid content was determined. The test revealed that the oil contained 44.81% linoleic acid and 33.40% oleic acid. Studies have shown that linoleic acid and oleic acid have a certain antioxidant capacity [25,26], but further studies are needed to prove the specific pathways and causes of the effect.

In our anti-fatigue studies, Yho-L, Yho-M and Yho-H significantly prolonged the weight-bearing swimming time in mice (*p* < 0.05), playing a role in anti-fatigue. HG was an important substance to provide sufficient energy for muscle contraction [27], and the test revealed that Yho-M and Yho-H significantly improved the content of HG in the liver tissue of mice after the exercise (*p* < 0.05), indicating that it improved the endurance of the organism. LA was a product of the body’s anaerobic enzyme function, formed after excessive exercise due to incomplete aerobic metabolism, and its accumulation not only increased fatigue but also caused damage on the cardiac system to some degree [28]. Our preparation of Yho-M and Yho-H significantly lowered the level of LA in mice (*p* < 0.05), suggesting that yellow horn may improve aerobic oxidative energy supply. BUN was a metabolite that broke down proteins and amino acids when the body stopped breaking down sugars and fats due to excessive exercise [29]. The experimental results showed that Yho-H significantly reduced the content of BUN (*p* < 0.05), and the effect was better than LPT, which was due to the yellow horn oil not needing to break down more protein and amino acids for energy replenishment. Therefore, the higher HG levels and the lower LA and BUN levels indicated that the body was more resistant to fatigue. In conclusion, the yellow horn oil had the effect of increasing endurance and acting as an anti-fatigue agent in mice.

The results of the antioxidant assay showed that Yho-H significantly increased GR content in mouse liver tissue (*p* < 0.05). GR was hydrogen-donated by the coenzyme NADPH, which catalyzed the oxidation of glutathione and the formation of reduced glutathione. Reduced glutathione could keep the sulfhydryl-containing enzyme in a reduced and active state [30]. Thus, Yho-H was beneficial in maintaining the integrity of the red blood cell membrane and preventing the oxidation of hemoglobin. It was notable that Yho-H was more effective than LPT in increasing SOD activity and decreasing MDA activity. MDA was the main by-product of lipid peroxidation and an important indicator of oxidative damage in cells [31]. SOD played an essential role in eliminating free radicals and protecting cells from damage [32]. In summary, higher GR levels were associated with stronger SOD activity, while weaker MDA activity indicated an increase in the body’s antioxidant capacity; it was shown that the yellow horn oil could play a role in scavenging free radicals. Therefore, it can inhibit the formation of lipid peroxidation products in the body, reduce the accumulation of oxygen free radicals and improve the anti-oxidant activity of animals.

## 4. Materials and Methods

### 4.1. Materials and Reagents

Yellow horn seeds were provided by Beijing Jintongfu Green Energy Technology Co., Ltd. (Beijing, China). CO_2_ (edible grade 99.999%) was obtained from Beiwen Gas Manufacturing Plant Co., Ltd. (Beijing, China). The 37 fatty acid methyl ester standards were purchased from ANPEL-TRACE Standard Technical Services Co., Ltd. (Shanghai, China).

Salad oil was obtained from Aceties Monterreal S.A. Rat maintenance pellet feed, conforming to GB 14924.3-2010, was purchased from Beijing Keao Xieli Feed Co., Ltd. (Beijing, China). Its Production License No. is SCXK (Beijing) 2018-0012, and it was freely ingested after autoclaving. Lentinan polysaccharide tablets (LPTs) were from Zhejiang Puloukangyu Natural Medicines Co., Ltd. (Jinhua, China). Their NMPA (National Medical Products Administration) approval number is Z20126215, and their shelf life is 24 months. Before being used, the tablet was ground into powder with a mortar, prepared with distilled water and mixed well with a stirrer. It was sealed and stored at 4 °C in the refrigerator until use. Physiological saline was provided by Shandong Kangning Pharmaceutical Co., Ltd. (Liaochen, China). The Lot No. is E10110809. It was stored in a sealed container at room temperature until it was directly used as a solvent for the solution required for the preparation of the kit.

The malondialdehyde (MDA) kit, superoxide dismutase (SOD) kit, lactic acid (LA) kit, glutathione reductase (GR) kit and hepatic glycogen (HG) kit were purchased from Nanjing Jiancheng Bioengineering Institute (Nanjing, China). The blood urea nitrogen (BUN) kit was purchased from Shanghai Rongsheng Biological Pharmaceutical Co., Ltd. (Shanghai, China). The above reagents were stored in a refrigerator at 4 °C and used for the determination of serum and liver samples.

### 4.2. Supercritical Fluid Carbon Dioxide (SF-CO_2_) Extraction of Yellow Horn Oil

The yellow horn oil was extracted from yellow horn seeds according to Zhang et al. [3]. Briefly, the yellow horn seeds were dried in a drying oven at 105 °C for 2 h and cooled at room temperature. Then, they were ground by a high-speed universal pulverizer (High-speed Universal Pulverizer Model FW100, Tianjin Teste Instruments Co., Ltd., Tianjin, China) passing through a 0.2 mm sieve. The sieved yellow horn seed powder was dried to constant weight, where the seed moisture content was 12.3%. An amount of 10 g of the powder was poured into the extraction kettle. The extraction was carried out using an SF-CO_2_ extractor (Supercritical Carbon Dioxide Extractor, Applied Separations, 930 Hamilton Street Allentown, Allentown, NJ, USA) for 30 min. The CO_2_ flow rate was maintained at 3.0 L/min. The extraction yield was calculated by the collection from the discharge port. Each group was extracted three times and the collections of the same group were combined. Extraction yield (%) was calculated and expressed on a wet-weight basis.

#### 4.2.1. Experimental Design

Single-factor tests analyzed the effects of extraction time, extraction temperature and extraction pressure on the extraction yield of the oil. When a factor was investigated, the other factors were set to fixed values, with the extraction pressure *p* = 35 MPa, extraction temperature T = 40 °C and extraction time t = 90 min. The factors and levels are shown in Table 3.

The following experiment was conducted after taking different factors into consideration and analyzing the results of the single-factor test. The extraction pressure (A), temperature (B) and time (C) were selected as the investigating factors, and three levels were taken for each factor. The extraction yield of the yellow horn oil was the investigating index, and the L_9_ (3^4^) orthogonal table was selected for the orthogonal design (Table 4). Because there are only 3 factors in this experiment, we made the last column of the L_9_(3^4^) orthogonal table an empty column.

#### 4.2.2. Fatty Acid Identification

We added 20 mL of n-heptane to 100 mg of yellow horn oil and then 8 mL of 2% methanolic sodium hydroxide solution to produce fatty acid methyl esters by saponification and esterification reactions. Fatty acids were analyzed using a Nexis GC-2030 gas chromatograph (GC) (SHIMADZU, Kyoto, Japan). The chromatograph was equipped with a CD-2560 (0.2 μm, 100 m × 0.25 mm) capillary column and a flame ionization detector (FID). Fatty acids were identified by comparing retention times to standards of 37 fatty acid methyl esters. Their contents are reported as relative percentages of individual fatty acids.

### 4.3. Studies on the Anti-Fatigue and Antioxidant Properties of Yellow Horn Oil

#### 4.3.1. Animals and Experimental Design

SPF-grade ICR strain mice: 60 males (starting mass 17.9 ± 1.0 g, 6 weeks old; mass at administration 22.9 ± 1.2 g, 6–7 weeks old), purchased from Beijing Vital River Laboratory Animal Technology Co., Ltd., Beijing, China. They were housed at 25 ± 1 °C, 50–60% humidity and maintained on a 12 h/12 h light/dark cycle, where they had free access to food and water during the adaptive rearing period. The animal procedures were performed in accordance with the Guidelines for Care and Use of Laboratory Animals of the Beijing University of Agriculture and the animal protocols used were approved by the Animal Ethics Committee of the Beijing University of Agriculture.

#### 4.3.2. Mice Administered by Batch Gavage

After 1 week of adaptive feeding, the above qualified ICR mice weighing 18–22 g were randomly divided into 5 groups of 12 mice each according to their weight, namely blank control group, positive control group (lentinan polysaccharide tablet 300 mg/kg), and extracted high-, medium- and low-dose groups (2.5, 5.0 and 10.0 mL/kg.bw/d in terms of crude oil content). Each dosing group was given the corresponding drug by gavage. The control group was given an equal amount of salad oil 10.0 mL/kg.bw/d by gavage once a day. In this case, the high dose of the extracted yellow horn oil was applied directly to the crude oil, while the medium- and low-dose groups were prepared by being diluted with salad oil, sealed and stored at 4 °C in the refrigerator until use.

#### 4.3.3. Weight-Bearing Swimming Test in Mice

The test mice were first grouped and labeled by the picric acid staining method. The animals were numbered in the order “group number + cage number + individual number”. The different groups were distinguished by different colored cage cards. The specific group number, number of animals, cage number and individual number are shown in Table 5.

The numbered mice were given the drug for 28 consecutive days, and the weight-bearing swimming test was performed 1 h after the last dose and this test was conducted after 9:00 am each day. The procedure used in this experiment was similar to that described by Huang et al. [33]. The mice used in the test were trained to swim for 30 min three times a week before the formal test and had adapted to the swimming environment at the time of the formal test. The mice were put into a swimming tank at a water temperature of 27 ± 0.5 °C and a water depth of 40 cm with a 5% lead skin weight on their tails. Physical exhaustion is defined as the inability to swim out of the water with the head submerged (for 10 s), and the time from their plunging into the water to their physical exhaustion is recorded as swimming time.

#### 4.3.4. Sample Collection and Parameter Determination

The mice were removed after swimming, all mice were killed by 95% CO_2_ asphyxiation and then blood and liver were collected immediately. All the experimental and care procedures used for animal euthanasia were performed in accordance with the relevant guidelines and regulations and were approved by the Committee on the Ethics of Animal Experiments of the Beijing University of Agriculture.

After their blood was collected from their eyes, the blood was centrifuged at 2000 rpm/min for 15 min after clotting. The serum was taken and frozen, and the livers were taken in triplicate, weighed (according to the different requirements of the three kits for HG, MDA and SOD) and placed in pre-prepared tubes for freezing. The serum and liver were stored at −80 °C.

The serum was used to measure the LA, BUN and GR; the liver was used to measure the HG, MDA and SOD. The serum BUN was measured by a fully automated biochemical analyzer (Hitachi 7060, Hitachi, Japan), and the other indicators were measured by kits. After the absorbance OD value was measured according to the instructions, the indicators were calculated according to the instructions.

### 4.4. Statistical Data Processing Methods

All results were expressed as mean ± the standard deviation (SD) plotted using Origin 2019b. Statistical data of three independent replicates were analyzed using SPSS statistical package 24.0 (SPSS Inc., Chicago, IL, USA). Data were subjected to two-way analysis of variance. Mean comparison was performed using the Duncan test at a significance level of 0.05 (*p* < 0.05).

## 5. Conclusions

The aim of this experiment was to extract the oil of the yellow horn seeds using the supercritical fluid CO_2_. The results of the single-factor and orthogonal tests showed that the optimum process conditions were 40 MPa extraction pressure, 50 °C extraction temperature and 120 min extraction time, in which the extraction yield was 31.61%.

The yellow horn oil obtained by SF-CO_2_ can prolong the time of weight-bearing swimming in mice, increase the content of HG and decrease the content of LA and BUN in mice. It was shown that the oil could promote exercise endurance in mice. Additionally, the yellow horn oil obtained by SF-CO_2_ could also decrease the content of MDA and increase the content of GR and SOD in the mice. It suggested that the oil effectively reduced the damage caused by the damage factors produced in the body at the same time. Therefore, yellow horn oil has certain anti-fatigue and antioxidant effects. This study laid the foundation for yellow horn and broadened its use. Further research should investigate the specific bioactive substances responsible for anti-fatigue and antioxidant effects.

## Figures and Tables

**Table 1:** (**a**). Results of the L_9_ (3^4^) orthogonal experiment. (**b**). Analysis of variance table.

(**a**)					
**Test Number**	**Factors**				**Extraction Yield/%**
	***A***	***B***	***C***	***D***	
1	1	1	1	1	22.65
2	1	2	2	2	25.72
3	1	3	3	3	24.02
4	2	1	2	3	24.58
5	2	2	3	1	26.87
6	2	3	1	2	25.42
7	3	1	3	2	25.90
8	3	2	1	3	24.41
9	3	3	2	1	31.61
*K*_1_	24.13	24.38	24.16	27.04	
*K*_2_	25.62	25.67	27.31	25.68	
*K*_3_	27.31	27.02	25.60	24.34	
*R*	3.18	2.64	3.15	2.71	
(**b**)					
**Source of Variance**	**Sum of Squared Deviations**		**Freedom**	***F*-Value**	**Significance**
*A*	124.27		2	26.65 *	*p* < 0.05
*B*	75.45		2	16.23	
*C*	134.89		2	23.32 *	*p* < 0.05
Error	17.99		2		
*F*_0.05_ (2, 2)	19			*F*_0.01_ (2, 2)	99

Note: extraction pressure (*A*), extraction temperature (*B*), extraction time (*C*), empty column (*D*). Note: “*” indicates significant factor influence.

**Table 2:** Fatty acid content of yellow horn oil.

Serial Number	Fatty Acids	Molecule Formula	Relative Content (%)
1	Butyric acid	C_4_H_8_O_2_	0.03
2	Palmitic acid	C_16_H_32_O_2_	4.82
3	Linolenic acid	C_18_H_30_O_2_	4.49
4	Linoleic acid	C_18_H_32_O_2_	44.81
5	Oleic acid	C_18_H_34_O_2_	33.40
6	Stearic acid	C_18_H_36_O_2_	1.30
7	Eicosapentaenoic acid	C_20_H_30_O_2_	0.70
8	Arachidonic acid	C_20_H_32_O_2_	0.10
9	Paullinic acid	C_20_H_38_O_2_	0.08
10	Arachidic acid	C_20_H_40_O_2_	0.08
11	Docosahexaenoic acid	C_22_H_32_O_2_	4.63
12	Erucic acid	C_22_H_42_O_2_	1.50
13	Nervonic acid	C_24_H_46_O_2_	4.06

**Table 3:** Table of Factor Levels for Single-Factor Tests.

Levels	Factors		
	Extraction Pressure (MPa)	Extraction Temperature (°C)	Extraction Time (min)
1	20	20	60
2	25	30	90
3	30	40	120
4	35	50	150
5	40	60	180

**Table 4:** Table of Factor Levels for Orthogonal Tests.

Levels	Factors			
	*A* Pressure (MPa)	*B* Temperature (°C)	*C* Time (min)	*D* Empty Column
1	30	30	90	1
2	35	40	120	2
3	40	50	150	3

Note: The empty column levels 1, 2 and 3 only indicate a virtual level and have no real meaning.

**Table 5:** Group markers for weight-bearing swimming test in mice.

Dose Group	Group Number	Animal Count	Cage Number	Individual Number	Cage Card Color
Control	1	6	01	1M0101~05	White
		6	02	1M0201~05	
LPT	2	6	01	2M0101~05	Red
		6	02	2M0201~05	
Yho-L	3	6	01	3M0101~05	Light green
		6	02	3M0201~05	
Yho-M	4	6	01	4M0101~05	Dark green
		6	02	4M0201~05	
Yho-H	5	6	01	5M0101~05	Blue
		6	02	5M0201~05	

Note: Blank control group (Control), lentinan polysaccharide tablet (LPT), yellow horn oil low-dose group (Yho-L), yellow horn oil medium-dose group (Yho-M), yellow horn oil high-dose group (Yho-H).

## Data Availability

The original contributions presented in the study are included in the article. Further inquiries can be directed to the corresponding author.
